# An environment for relation mining over richly annotated corpora: the case of GENIA

**DOI:** 10.1186/1471-2105-7-S3-S3

**Published:** 2006-11-24

**Authors:** Fabio Rinaldi, Gerold Schneider, Kaarel Kaljurand, Michael Hess, Martin Romacker

**Affiliations:** 1 Institute of Computational Linguistics, IFI, University of Zurich, Switzerland; 2 Novartis Pharma AG, Basel, Switzerland

## Abstract

**Background:**

The biomedical domain is witnessing a rapid growth of the amount of published scientific results, which makes it increasingly difficult to filter the core information. There is a real need for support tools that 'digest' the published results and extract the most important information.

**Results:**

We describe and evaluate an environment supporting the extraction of domain-specific relations, such as protein-protein interactions, from a richly-annotated corpus. We use full, deep-linguistic parsing and manually created, versatile patterns, expressing a large set of syntactic alternations, plus semantic ontology information.

**Conclusion:**

The experiments show that our approach described is capable of delivering high-precision results, while maintaining sufficient levels of recall. The high level of abstraction of the rules used by the system, which are considerably more powerful and versatile than finite-state approaches, allows speedy interactive development and validation.

## Background

Information overload is one of the most widely felt problems in our modern society. Individuals have access to a previously unimaginable flood of new information and professionals are confronted in their daily activities with a cornucopia of relevant results. Especially for biomedical scientific literature, there is a pressing need for an efficient approach to access and extract information, in a format that can be easily assimilated by humans or further processed by other automated tools.

Most of the biomedical literature is currently accessible through PubMed , which offers a keyword-based search over the published articles. Existing domain knowledge is gradually systematized into manually compiled ontologies, such as the Gene Ontology , or pathway databases, such as KEGG . The maintenance of such resources is a labour intensive process. Besides, there might be a significant time lag between the publication of a result and its introduction into such databases. Relevant articles have to be selected and accurately read by human experts looking for the core information. This process is usually referred to as *curation *of the article.

A (partial) automation of this activity is therefore highly desirable. The first step is the identification of all biological relevant entities (genes, proteins, diseases, etc.). This task has been addressed quite extensively by the research community, as witnessed by events such as BioCreAtIvE . The task is made particularly difficult by the high ambiguity of the entity names in this domain: in addition to a high degree of polysemy and synonymy, very common words can be used as names of entities [1].

We have chosen to skip this problem, in order to focus on the next step, which is the detection of the possible interactions among the entities mentioned in a document. Therefore for this experiment we use an existing manually annotated corpus. However, in [2] we describe how our system can cope with an automatically annotated corpus using an external tool for the detection of the domain entities and terminology.

Tools capable of automatically constructing pathways from published articles are starting to appear, both as research prototypes [3], and as commercial systems [4]. Given the complexity of the task, typically only a few semantic relations are output, for which the confidence is very high, based on the analysis of large quantities of documents.

Our aim is to show how a deep-linguistic approach can be used in a Text Mining application, offering high-precision relation extraction, while at the same time retaining a high recall. The results are validated on a richly annotated resource: the GENIA corpus.

After briefly introducing the GENIA corpus in section "Corpus Analysis", we detail the processing steps that have been adopted in order to extract a rich set of linguistic and domain-specific information. Section "Relation Mining" shows in particular how the intermediate results of data analysis are used in the Relation Mining task. Section "Evaluation" describes the evaluation of the results. Section "Related Work" surveys related work. We conclude by describing plans for future work in section "Conclusions and Future Work".

## Methods: Corpus analysis

GENIA [5] is a corpus of 2000 Medline abstracts which have been *manually *annotated by domain experts with biological entities from the GENIA Ontology. The base abstracts were selected from Medline using the keywords "Human", "Blood Cells", and "Transcription Factors". Using near-perfect GENIA annotation enables us to simulate a situation in which future, mature term recognition is used, allowing us to focus on the impact of parsing and relation mining techniques. This section describes the approach taken in analyzing the input corpus. The tools that we use for such processing steps are organized into a Natural Language Processing pipeline including a fast, deep-linguistic statistical dependency parser. The pipeline and the parser are described separately below. The final result of the analysis process is a set of dependency relations, which are encoded as (sentence-id, type, head, dependent) tuples. This is a format which is well suited for storage in a relational DB or for analysis with Data Mining algorithms. In the case of GENIA, we make the entire set of our annotations freely available for research purposes on our web site at . The dependency relations, together with intermediate results of the pipeline (tokens, terms, chunks, sentences) are stored in a Knowledge Base (KB), which can then be queried by a separate module, described later in section "Relation Mining".

### The NLP pipeline

The pipeline [6] performs a sequence of processing tasks, described below. In the case of GENIA, some of these steps (e.g. tagging, terminology detection) are not necessary – and are automatically skipped – because the relevant information is already provided in the Corpus.

1. Sentence splitting by MXTERMINATOR [7]

2. Tokenization by the Penn Treebank tokenizer

3. Part-of-speech tagging by MXPOST [8]

4. Lemmatization by morpha [9]

5. Term extraction by matching the token stream against existing term lists from biomedical ontologies

6. Replacing of multi-word terms with their heads

7. Noun and verb group chunking by LTCHUNK [10]

8. Detection of chunk heads by a simple pattern matching over the part-of-speech tags of the tokens

9. Dependency parsing

**Figure 1:**
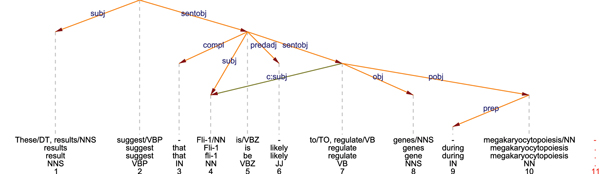
**Example of Dependency Tree**. Tree of dependencies for a GENIA sentence, along with other linguistic annotations. Notice the additional deep-linguistic "control" subject dependency between token 7 and 4.

When the pipeline finishes, each input sentence has been annotated with additional information (figure 1 shows a graphical example), which can be briefly summarized as follows: sentences are tokenized and their borders are detected; each sentence and each token has been assigned an ID; each token is lemmatized; tokens which belong to terms are grouped; each term has a normal-form and a semantic type; tokens and terms are grouped into chunks; each chunk has a type (NP or VP) and a head token; each sentence is described as a syntactic dependency structure; each dependency occurs between two tokens and has a type. All this information is represented as a set of predicates and stored into the KB of the system, which can then be queried using the methodology described in section "Relation Mining".

### Parsing the corpus

We use a robust, deep-syntactic, broad-coverage probabilistic Dependency Parser [11], which identifies grammatical relations between the heads of chunks, chunk-internal dependencies, and the majority of long-distance dependencies [12].

The output is a hierarchical structure of syntactic relations: functional dependency structures, represented as the directed arrows in figure 1. [13] discusses that this representation is very similar to the f-structure known from Lexical-Functional Grammar. The parser uses a hand-written grammar expressing linguistic competence and a statistical language model that calculates lexicalized attachment probabilities, thus expressing linguistic performance.

The supervised model based on Maximum Likelihood Estimations (MLE) extends on [14] and calculates the probability of finding a specific syntactic relation *R *(such as subject, sentential object, etc.) given the lexical head (*a*) and dependent (*b*) at the distance (*δ*) in chunks between them (further details can be found in [11]).



The parser expresses distinctions that are especially important for a predicate-argument based deep syntactic representation, as far as they are expressed in the Penn Treebank training data [15]. This includes PP-attachment, most long-distance dependencies, appositions, relative clause anaphora, participles, gerunds, and argument/adjunct distinctions.

The parser is very robust and has been applied to parsing large amounts of text data, including the 100 Million word British National Corpus . It does not always deliver a parse spanning the entire sentence, however it never fails completely, always delivering at least partial structures. Table 1 shows a comparison of two evaluations performed using the parser. For the first result, we apply the standard 500 sentence test set for dependency parsers, GREVAL [16], in order to assess its performance on general text. The results obtained are comparable to other parsers [16-18]. For the second result, we use a random set from the GENIA corpus in order to assess its performance on the biomedical domain. We have randomly selected 100 sentences from the GENIA corpus, which we have manually annotated for the syntactic relations that the parser can detect. Our results suggest that parsing performance on biomedical texts can be similar or better to the one on general text, for the following reasons:

• We have observed that verbs and prepositions, which are especially important for the lexicalized disambiguation, vary far less between general text and the biomedical domain than nouns.

• A class of nouns that varies considerably in the biomedical domain are relational nouns. They are syntactically marked because they can have several PP arguments. Biomedical relational nouns like *overexpression *or *transcription *are absent from the Penn Treebank or rare. We use an unsupervised approach based on [19] to learn relational nouns from Medline.

• Chunkers often make errors on domain-specific multi-word terms, part-of-speech taggers typically make errors on gene names. High-quality domain entity recognition is therefore key to successful parsing in the biomedical domain, as we show in [20].

## Methods: Relation mining

Our approach to relation mining is based on 3 levels of rules. On the first level, we exploit simple *syntactic patterns *detected in the data. On the second level we combine various patterns into a single *semantic rule*, which normalizes many possible syntactic variants (e.g. active, passive, nominalizations). On the third level we combine semantic rules with lexical and ontological constraints to obtain very specialized queries that can detect a given domain-specific relation, as specified by the user.

The final goal is to extract and present all relations that are needed to construct complete pathways, since this is the representation that domain specialists eventually like to work with.

### Syntactic queries

We have written a set of syntactic rules that capture some of the most important syntactic phenomena, as in the example below, which encodes the passive case:

synRel(passive, [X1, X2, X3],

   [dep(subj, X2, X1), dep(pobj, X2, X3),

      dep(prep, X3, By), pos(X2, 'VBN'),

      lemma(By, ['by', 'through', 'via'])]).

To simplify the process of detecting interesting patterns, the expert can make use of a web interface (see ) which allows to interactively construct a pattern, see the results of applying it over a pre-analyzed corpus, and (if the user is satisfied with the result) save it as a rule.

Syntactic rules capture general linguistic phenomena and as such are highly reusable across different domains. Simpler rules can easily be combined into more complex ones – thus making the system more modular.

### Semantic queries

The next step is then to combine different syntactic patterns to yield a *semantic rule*. A generic relation between two arguments (A and B), mediated by a verb or an equivalent relational noun (H), is most commonly expressed by one of the following patterns:

semRel(xrel([H, A, B]), active([A, H, B])).

semRel(xrel([H, A, B]), passive([B, H, A])).

semRel(xrel([H, A, B]), nominalization([H, B, A])).

While in the active case (e.g. *"A inhibits B"*) the subject of the sentence expresses the agent (A) and the direct object expresses the target (B) of the relation, in a passive sentence (e.g. *"B is inhibited by A"*), the agent is expressed by a prepositional phrase (e.g. *"by A"*), while the subject expresses the target of the relation. In both these cases, the main verb of the clause (*inhibit*) expresses the relation (H) between the arguments. In a nominalization (e.g. *"The inhibition of B by A"*) the relation is expressed by a relational noun, while the two arguments are expressed by prepositional-phrase attachments. The argument A will be referred to as the agent, B as the target, adopting the terminology used in [21]. The argument H – the 'head' verb – defines the type of the relation (e.g. *"activate"*).

The equivalence rules expressed above allow the user to formulate powerful queries which capture all the defined variants of the given configuration. For example, the query below returns all the sentences containing a control relation, where A and B are instantiated respectively by the agent and the target of the relation:

applyRel(xrel(['control', A, B]))

Alternatively, it is possible to phrase a query which seeks all relations where a given entity participates, e.g.

applyRel(xrel([H, A, 'NF-kappa B']))

This query returns all the relations where *"NF-kappa B" *is involved as a target (e.g. *"In T cells, NF-kappa B is activated upon cellular treatment by phorbol esters and the cytokine tumor necrosis factor alpha."*).

The 3-argument relation discussed above can easily be extended with additional arguments. For example in many cases it is important to be able to detect the polarity (which refers to positive vs negative cases, e.g. *"A does not inhibit B"*) and the modality (which refers to some property which restricts the validity of the asserted relation, e.g. *"A might inhibit B"*) of the relation. We have implemented patterns and rules that can cope with these cases, but they have not been evaluated yet, and therefore for the present study they are not further considered.

### Ontology-based queries

If a domain Ontology is available, our system can make use of it in the query process, by using the types as restrictions for the arguments. If the types are not structured into a taxonomy, this results in the extraction of all relations where the arguments satisfy exactly the given restriction.

However, if an Ontology is available (by "Ontology" in this context we mean simply a taxonomical organization of domain specific concepts) we can extend the interpretation of the type restriction to mean not only the objects that directly match the given type, but also those that have a type subsumed by it. This is possible in the case of the GENIA corpus, because the entity annotations have been created according to the types defined in the GENIA Ontology .

For example, the following restrictions can be used in a query in order to limit the type of the agent to be "protein_molecule":

applyRel(xrel(['control', type: ' G#protein_molecule',_])).

While the following allows it to be "amino_acid" which is a more generic term, according to the Genia Ontology:

applyRel(xrel(['control', type: ' G#amino_acid',_])).

Because "amino_acid" is a supertype of "protein_molecule", according to the given ontology (although this might appear incorrect to a domain expert), the results of the latter include (and expand) the results of the former.

### Additional features

**Figure 2:**
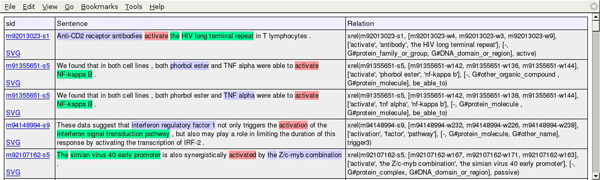
**Sample Output**. Sample output for the 'activate' relation.

As the set of rules is gradually enriched, so are the possible lexico-syntactic variants that can be captured. For example in figure 2, the last of the examples shown is a case of a complex rule designed to capture the pattern *"A triggers the H of B"*, where H represent a nominalized verb (*activation, regulation, etc.*). Similar complex rules have been designed, e.g. for *"under the control of"*, *"involved in"*, *"be able to" *etc. Such complex rules can be designed by listing all the syntactic and lexical constraints, or alternatively can be constructed combining syntactic and semantic rules, as in the example below:

domRel(trigger3([H, A, B]),

            [xrel(['trigger', A, H]),

            nominalisation(H, Prep),

            depRel([H, B, Prep])]).

We refer to relations defined at this level as *domain relations *as they rely on lexical constraints which are typical of a given domain. The user query can happen at each one of the 3 levels.

We have designed the search algorithm so that a few basic syntactic patterns are expanded by default. This includes for example the case of conjunctions, as it can be seen in one of the examples shown in figure 2. The main focus in the design of our Text Mining environment has been on ease of use, and therefore we provide utilities for debugging and visualization. For example, in figure 2 it can be seen that each result bears the name of the rule that generated it (last argument of the third column). This allows immediate detection of problems and their quick correction. We also provide a "visual diff" facility that shows in the same graphical format the matches that have been acquired or lost as a consequence of the addition of a new rule.

## Results: Evaluation

The approach followed for creating rules starts from a set of relations that are of particular interest in this domain, such as: *activate*, *bind*, *block*, *regulate*, *control*, *express*. The rule developer is offered a view over all the sentences that might include one of the selected relations (detected using as keywords the verbs that express such relation and all their grammatical inflections). While rules are being developed, the view changes, signaling which sentences are being captured by which rules. New rules can make use of rules defined at lower levels, some of which remain stable across different applications.

In order to simplify the process of evaluation and shorten the development cycle, we have created visualization tools (based on XML, CSS and CGI scripts), that can display the results in a browser. The sentences which contain one of the relations identified by the query are collected and displayed sequentially in a XHTML page, where the arguments of the relation are marked with a predefined color scheme. In this way it is immediately obvious to the user whether the tools have done a proper job, or a mistake has been introduced at some stage of processing (see figure 2).

When the coverage is satisfactory, it is possible to proceed to an evaluation, like the one described in this section, which however refers to a particular 'snapshot' of the system at a given point in time. Further extensions to the rules are likely to lead to improved results.

Table 2 shows the results obtained on a subset of the relations extracted by the system. We asked the domain experts to evaluate each relation and each argument of the relation, and mark them according to the following guidelines:

Y if the relation is correct and biologically significant, treat as correct

A if the relation is correct and biologically significant, but includes too much or too little information (for example because an informative PP is not highlighted or a non informative PP is highlighted), treat as correct

P if the relation appears correct, but an anaphora needs to be resolved, treat as incorrect

N if the relation is completely wrong, treat as incorrect

For both arguments the exact results [Y] are above 50%. When we slightly relax the precision criteria and include the cases [A] where the argument has been correctly identified, but incorrectly expanded, precision jumps to about 90%. The [A] cases can be considered almost correct, as it easy (by simply examining the highlighted arguments) to detect the correct boundaries of the argument, should that be required. Unresolved pronouns [P] need a reader to deal with a substantially larger context (e.g. "this protein", referring to a protein mentioned in the previous sentence). Our system does not yet include an anaphora resolution algorithm, therefore we have decided to report these cases as incorrect.

In the absence of a gold standard, only approximative recall values can be reported. In [2] we report a value of 40% for a measure that we call "worst-case recall", which basically implies that our actual recall is at least as good as this value. On a smaller subset of the corpus we actually measured a recall value of 60%. Using the recall obtained on the development set and the measurable coverage (how many cases of "potential" relations the system actually detects) on the test set, we can estimate the value of recall on the latter (if we make the assumption that the verb-specific ratios between coverage and recall are similar across two corpora of the same genre). By extrapolation we get the approximative recall results in table 3. The extrapolation from the coverage with both agent and target (2 dep) and the extrapolation from the coverage with either agent or target (1 dep), based on the coverage to recall ratios, delivers 2 estimates, which are shown as a range in the third column.

An analysis of 46 precision and recall errors from a subset of the pattern development corpus reveals the following sources of errors, with numbers of cases in brackets: conjunction or apposition parsing or expansion error (15), parsing span too small (14), other parsing error (5), chunk-internal relation detection error (4), part-of-speech tagging and chunking error (4), syntactic phenomena not covered by grammar (2), pattern errors (2).

## Related work

The task of relation extraction can be performed at different levels of complexity. The systems that deal with this task can be broadly classified in three categories, according to the amount of linguistic information brought to bear on the problem.

The simplest approach is based on the recognition of **surface patterns**, i.e. sequences of words or PoS tags that identify a particular type of interactions. Such patterns can be manually written, or, more frequently, automatically induced from a manually annotated corpus. An example of this approach is given by [22]. While surface patterns are easy to learn and computationally efficient, they fail to generalize on even the most obvious linguistic variations. Therefore many systems resort to **shallow parsing **approaches, which typically detect the main constituents of the sentences, without building a complete syntactic analysis. Typically such systems make use of external resources, such as domain Ontologies, in order to detect the most likely combination of the constituents of the sentences, based on their semantic types. Some examples are [23-26].

The most challenging approaches are those based on **full parsing, **which attempt to build a complete syntactic structure for each sentence in the corpus. Traditionally such approaches have been limited by the brittleness of the existing parsers. However, recent advances in probabilistic-based parsing allow to overcome such limitations and render such approaches competitive. We discuss below a few systems that make use of full parsing approaches for the analysis of biomedical literature.

The Tsujii group uses an HPSG parser [27] to identify Predicate-Argument Structure, using a domain-independent approach. They apply a pattern extraction algorithm to induce rules from a development corpus that are then applied to a test corpus. The results are relatively good (33% F-Measure) for an approach which aims at avoiding manual construction of rules. More recently, their HPSG parser has been applied to the entire Medline. A demonstration of a relation extraction application is available at .

MedScan [28] makes use of a syntactic parser (which typically yields a large number of analyses for each sentence) and a semantic processor which transforms each syntactic tree into a corresponding semantic tree. Information extraction rules are then used to prune the large number of trees and extract from them the information of interest. Their system is impressive, but the syntactic analysis is not robust (they report 34% coverage). On the task of extracting human protein interactions they report 91% precision and 21% recall.

GENIES [29] is a system, based on a DCG grammar, which processes biomedical literature in order to detect information about cellular pathways. The system, which uses not only syntactical but also semantical constraints, attempts to obtain a full parse in order to achieve high precision, but often backs off to partial parsing to improve recall.

The "Learning Language in Logic" challenge (LLL05) [21] recently has seen systems competing on the task on inducing IE rules to be used to extract information on gene/protein interactions, in particular focusing on interactions between protein agents and their gene targets in *Bacillus subtilis*. Among the systems that participated, the experience of [30] shows that an approach based on syntactic information can deliver very good results. A different approach, based on learning simple surface patterns (which encode only lexical information, word order and PoS tags) is followed by [31]. Interestingly, both approaches, although based on very different assumptions, delivered good results and were the most successful in the competition.

## Conclusions and future work

In this paper we have presented an approach aimed at supporting the process of extraction of core relational information from scientific literature in the biomedical domain. We have based our experiments on an extended version of the manually-annotated GENIA corpus. We have shown how the user can quickly and efficiently develop and test new patterns over a medium-sized corpus. Examples of quite sophisticated patterns have been illustrated. The approach is validated by an evaluation based on the GENIA corpus. The parser described in this paper, the relation mining system, and the evaluation dataset, can be obtained by contacting the authors. A web demo, with limited functionalities, can be accessed at .

The same approach could be applied to any corpus where entities have been annotated using types organized in a taxonomical structure. In the case of biomedical literature, more complex Ontologies could be used, for example the Gene Ontology .

We are currently setting up a framework for an intensive collaboration, which will allow us to apply the approach described in this paper to non-annotated corpora, using term recognition tools at Novartis. Since 2001, Novartis has information extraction and text mining applications in place that are used by hundreds of associates [32]. The applications consist mainly of a huge knowledge portal that comprises more than 40 external and internal data repositories. Additionally, the Computational Knowledge Management and Text Mining unit at Novartis supports a number of custom tailored text mining solutions for disease areas and pipelines.

One of the core components is an annotator that we intend to apply for the entity recognition task. We envisage to apply our approach to a larger corpora of full-text journal articles where Novartis has full access to 5 volumes of more than 200 journals available in electronic form. One of the advantages of the Novartis annotator is that it is built on a huge terminology with more than 1 Million terms. The terms contain gene names, targets, modes of action, diseases, geographic locations, products and companies. Furthermore, Novartis will allocate resources for manual annotation and evaluation of the results. The interaction with researchers who work on pharmaceutical topics will clearly provide very valuable feedback. This will help us to better customize our rules and to evaluate the quality of our approach in an iterative manner. A long-term goal is the combination of the results into complex pathway networks, which can then be presented graphically to the users.

## Figures and Tables

**Table 1:** Evaluation on Carroll's test suite on subj, obj, PP-attachment and subordinate clause relations.

GREVAL	Subject	Object	noun-PP	verb-PP	subord. clause
Precision	92.4%	89.1%	74.4%	72.4%	68.2
Recall	81.0%	83.9%	65.5%	84.8%	n/a

GENIA100	Subject	Object	noun-PP	verb-PP	subord. clause

Precision	90.0%	94.1%	83.3%	81.7%	71.1%
Recall	86.2%	94.9%	81.9%	84.2%	75.0%

**Table 2:** Analysis of precision for selected relations over GENIA

	agent				target			
	Y	A	P	N	Y	A	P	N

activate	72	64	5	8	77	54	8	10
bind	36	18	1	8	39	18	1	5
block	3	0	0	0	1	1	0	1
TOTAL	111	82	6	16	117	73	9	16
	52%	38%	3%	7%	55%	34%	4%	7%
	correct 90%		incorrect 10%		correct 89%		incorrect 11%	

**Table 3:** Estimate of recall. Extrapolated percentages are in boldface

Corpus	Relation	Recall	Coverage (at least 1 dep)	Coverage (2 dep)
ATCR (observed)	control	60%	106 out of 12 9	59 out of 129
	regulate	60%	116 out of 161	58 out of 161
GENIA (estimated)	control	**38 – 41%**	304 out of 541	155 out of 541
	regulate	**50 – 65%**	887 out of 1125	339 out of 1125
